# Assessing Quality of Life in Genital Lichen Sclerosus: The Role of Disease Severity and Localization—A Swedish Prospective Cohort Study

**DOI:** 10.3390/medsci13030111

**Published:** 2025-08-05

**Authors:** Filippa Lundin, Cassandra Jeppsson, Oliver Seifert, Georgios Kravvas, Sandra Jerkovic Gulin

**Affiliations:** 1 The Faculty of Medicine and Health Sciences, Linkoping University, 581 83 Linkoping, Swedencasje074@student.liu.se (C.J.); 2 Department of Dermatology and Venereology, Ryhov County Hospital, 553 05 Jonkoping, Sweden; oliver.seifert@liu.se; 3 Division of Cell Biology, Department of Biomedical and Clinical Sciences, Linkoping University, 581 83 Linkoping, Sweden; 4 Department of Dermatology, University College London Hospitals NHS Foundation Trust, London NW1 2BU, UK; georgios.kravvas@nhs.net; 5 Department of Medicine, University College London, London NW1 2BU, UK

**Keywords:** lichen sclerosus, lichen sclerosus score, quality of life

## Abstract

Introduction: Lichen sclerosus (LSc) is a chronic, progressive, inflammatory skin disease that primarily affects the anogenital region in both sexes and across all age groups. Aim: To investigate the relationship between quality of life (QoL) and disease severity, as measured by a newly developed Lichen Sclerosus Score (LSc score), with respect to anatomical site before and after 12 weeks of treatment. Methods: A total of 136 patients diagnosed with LSc (88 men, 48 women) were enrolled between March and September 2022. Patients were clinically evaluated using the LSc score and completed the Dermatology Life Quality Index (DLQI). Treatment was individualized based on clinical findings and history. At 12 weeks, both clinical assessment and DLQI were repeated. Results: LSc scores significantly decreased following treatment (*p* < 0.001), except in the female subgroup. In men, LSc scores were strongly correlated with DLQI scores both before (r = 0.709; *p* < 0.001) and after (r = 0.492; *p* < 0.001) treatment. Among women, a significant correlation was found only before treatment (r = 0.457; *p* < 0.001). Significant associations were identified between LSc score and DLQI items 1, 8, and 9 in men and the overall cohort. No statistically significant differences in LSc scores or DLQI were observed across anatomical sites after correction for multiple comparisons. Conclusions: Disease severity in genital LSc is closely associated with QoL impairment. This is, to our knowledge, the first study to examine the correlation between a clinical severity score and DLQI. While anatomical site did not significantly affect scores, certain sites may have a disproportionate impact, underscoring the complex ways in which LSc affects patients’ lives.

## 1. Introduction

Lichen sclerosus (LSc) is a chronic, progressive, inflammatory dermatosis that primarily affects the anogenital region. It can significantly impair urinary and sexual function [1]. Symptoms vary widely, ranging from asymptomatic cases to severely distressing presentations [2,3,4,5]. Diagnosis is largely clinical, although biopsy may be necessary in cases of diagnostic uncertainty or suspected neoplasia [3,6]. Clinical signs include rashes, sclerosis, adhesions, and structural changes such as effacement or the coronal sulcus and labia. Typical histological signs of MGLSc include epidermal atrophy, oedema and hyalinization of the superficial dermis. Deep to the hyalinized zone, a band-like lymphohistiocytic infiltrate can also be seen [3].

Prevalence estimates range from 1:60 to 1:1000 in the United States, with a recent Swedish study reported an incidence of 80.9/100,000 person-years [7,8]. Underdiagnosis is common, with up to one-third of cases remaining asymptomatic [9,10,11]. LSc can affect individuals of any age or sex, but is reported more frequently in females, with reported female-to-male ratios ranging from 3:1 to 10:1 [10,12].

Several etiological factors have been proposed, including genetic predisposition, immune dysregulation, and environmental influences [4,6,13,14]. Increasing evidence suggests that chronic exposure of vulnerable tissues to trapped urine droplets may play a key role [15,16,17]. Disruptions in the genital microbiome may also contribute to disease development [16,17,18,19,20].

Delayed diagnosis is frequently attributed to nonspecific symptoms, patient hesitancy, and fragmentated care across specialties [2,21,22,23]. If left untreated, LSc can lead to anatomical distortion, urinary and sexual complications, and an elevated risk of carcinogenesis [2,5,9,10]. While the psychosocial impact of LSc is increasingly recognized, studies exploring its effect on sexual health remain limited. One study reported sexual dysfunction in 83% of women with LSc, with nearly 40% experiencing depressive symptoms [24,25].

First-line treatment consists of ultrapotent topical corticosteroids, soap substitutes, and barrier preparations [5]. For treatment-resistant disease, circumcision should be considered in men, and systemic immunomodulation in women [5,26]. Given the heterogeneity of clinical presentations, various severity scales have been proposed. The Clinical Lichen Sclerosus Score (CLISSCO), introduced in 2020, combines symptoms and clinical signs but was developed specifically for female patients. Although validated scoring systems are essential for consistent assessment and monitoring, they remain underdeveloped, particularly for male patients [2,3,4,23,27].

Moreover, little is known about how disease involvement at different anatomical sites influences QoL or sexual function, particularly in men. Understanding the relationship between site-specific involvement and disease burden may inform more tailored and effective management strategies.

### Primary Aim of the Study

This study aimed to assess the relationship between subjective and objective measures of disease severity in genital LS. Specifically, we compared Dermatology Life Quality Index (DLQI) scores with a new modified Lichen Sclerosus Score (LSc score) across different anatomical sites, both before and after 12 weeks of treatment. The goal was to improve our understanding of QoL impairment in LSc and support the development individualized management strategies, particularly given the associated risk of malignant transformation.

## 2. Materials and Methods

### 2.1. Study Design and Population

This was a prospective cohort study conducted at the Department of Dermatology and Venerology, Ryhov County Hospital, Jönköping, Sweden. A total of 184 adult patients (≥18 years) with clinically diagnosed genital LSc were recruited between March and September 2022. Diagnosis was based on characteristic clinical features such as white plaques, atrophy, hypopigmentation, fine wrinkling, scarring, adhesions, and anatomical effacement. Patients were also concurrently enrolled in a parallel study investigating the skin microbiome in LSc.

Exclusion criteria are listed in Table 1. These included current pregnancy, recent use of certain medications, prior circumcision for LSc, and inability to provide informed consent. All participants provided written informed consent and retained the right to withdraw from the study at any time.

### 2.2. Data Collection

All patients underwent two study visits: at baseline and at 12 weeks. At each visit, they completed the DLQI and underwent a clinical evaluation using the modified LSc score (Table 2). Clinical signs were scored on a 3-point scale: 0 (absent), 1 (moderate), and 2 (severe). Treatment was prescribed according to clinical findings and medical history, following standard protocols at the study center.

Treatment regimens included both topical and systemic options. Topical corticosteroids (e.g., clobetasol) were applied once daily for four weeks, then every other day for the following four weeks, and twice weekly during the final four weeks. Adjunct therapies included barrier creams and soap substitutes. Men were evaluated for potential circumcision. Severe LSc in women was managed with systemic agents such as methotrexate or hydroxychloroquine, or in extreme cases with a 12-week course of oral Prednisolone (10 mg daily).

At the follow-up visit (week 12), both the DLQI and LSc score assessments were repeated. Of the 184 initially recruited patients, 136 (88 men and 48 women) completed both assessments and were included in the final analysis.

### 2.3. Dermatology Life Quality Index

The DLQI is a validated patient-reported outcome measure widely used to assess the impact of dermatologic conditions on quality of life (QoL) [28,29]. It comprises 10 questions evaluating symptoms (Q1), emotions (Q2), daily activities (Q3 and Q4), leisure (Q5 and Q6), work or school (Q7), personal relationships (Q8 and Q9), and treatment (Q10). Each item is scored on a 4-point scale (0–3), yielding a total score ranging from 0 to 30. Higher scores indicate greater impairment of QoL.

A total score above 10 is generally interpreted as a significant impact on the patient’s QoL. The questionnaire refers to experiences during the preceding week (see Supplementary File S1 for the full questionnaire).

### 2.4. Lichen Sclerosus Score

The modified LSc score is a composite instrument that evaluates both patient-reported symptoms and physician-assessed clinical signs. It includes eight items: four symptom-based (“pruritus,” “burning,” “soreness,” and “pain during intercourse”) and four sign-based (“erosions,” “sclerosis,” “erythema,” and “hypopigmentation”). Each item is graded on a 3-point scale: 0 (absent), 1 (moderate), and 2 (severe), producing a maximum total score of 16 (Table 2). Higher scores reflect more severe disease.

Anatomical sites of involvement were also recorded using schematic diagrams, with specific locations categorized as shown in Table 3. All clinical assessments were conducted by a single dermatologist to minimize inter-rater variability.

### 2.5. Ethical Considerations

The study was approved by the Swedish Ethical Review Authority on 15 November 2021 (reference number: 2021-05590-01). All participants provided written informed consent prior to inclusion, and participation was voluntary with the right to withdraw at any time.

### 2.6. Statistical Analysis

All statistical analyses were conducted using SPSS (IBM Corp., Armonk, NY, USA, Version 29.0.2.0) and Microsoft Excel (Microsoft Corp., Redmond, WA, USA, Version 16.96.1), with the exception of correlation comparison tests, which were performed using an online calculator for Fisher z-transformed coefficients.

Descriptive statistics were generated for age, total LSc scores, and DLQI scores before and after treatment. Normality was assessed using the Kolmogorov–Smirnov and Shapiro–Wilk tests, which confirmed non-normal data distribution.

To evaluate changes in LSc scores over time, the Wilcoxon signed-rank test was used. Differences between male and female patients were assessed using the Mann–Whitney U test. For comparisons of LSc and DLQI scores across anatomical sites, the Kruskal–Wallis test was employed, followed by Bonferroni-corrected post hoc analysis where appropriate.

Spearman’s rank-order correlation was used to assess associations between total DLQI and total LSc scores both before and after treatment. A z-test was applied to compare correlation coefficients between subgroups using Fisher z-transformation [30,31].

Additional Spearman correlations were conducted to explore the relationships between total LSc scores and age, and between total LSc scores and selected DLQI items (questions 1, 8, and 9), stratified by sex and timepoint (pre- and post-treatment).

## 3. Results

### 3.1. Cohort Characteristics

A total of 136 patients completed both study visits and were included in the final analysis. This cohort comprised 88 men and 48 women, aged 18–87 years. The median age for the entire cohort was 62 years (range: 18–87). Men had a median age of 59 years (range: 18–86) and women had a median age of 64 years (range: 31–87). Demographic details are summarized in Table 4.

### 3.2. Lichen Sclerosus Score (LSc Score)

**Figure 1:**
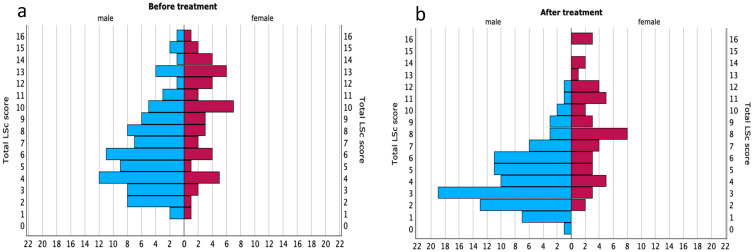
Distribution of lichen sclerosus scores before and after treatment, stratified by sex. Population pyramids illustrating the distribution of total LSc scores (range: 0–16) among male (n = 88) and female (n = 48) patients at (**a**) baseline and (**b**) after 12 weeks of treatment.

Median LSc scores (range: 0–16) before and after treatment are shown in Table 5 and Figure 1. At baseline, the overall median LSc score was 7 (interquartile range (IQR): 4–10), which decreased to 5 (IQR: 3–8) after 12 weeks. This reduction was statistically significant (*z* = −4.357; *p* < 0.001).

Among men, the median LSc score decreased from 6 (IQR: 4–9) to 4 (IQR: 3–6) following treatment, also reaching statistical significance (z = −4.568; *p* < 0.001). In contrast, although women’s scores declined from 10 (IQR: 6–13) to 8 (IQR: 5–11), this reduction was not statistically significant (*z* = −1.413; *p* = 0.158) (Table 5).

Comparisons between sexes showed that women had significantly higher LSc scores than men both before (*z* = −3.776; *p* < 0.001) and after (*z* = −5.784; *p* < 0.001) treatment.

No significant correlations were found between age and LSc score, either in the total population or when analyzed separately by sex.

### 3.3. LSc Score in Relation to DLQI

There was a strong, statistically significant correlation between the total DLQI score and the total LSc score before treatment (*r* = 0.633, *p* < 0.001). This association was stronger in men (*r* = 0.709, *p* < 0.001) than in women (*r* = 0.457, *p* < 0.001), with the difference between the two correlation coefficients reaching statistical significance (*z* = 2.12, *p* = 0.03).

After 12 weeks of treatment, a significant correlation remained between DLQI and LSc score in the overall cohort (r = 0.505, *p* < 0.001). When stratified by sex, the correlation persisted in men (r = 0.492, *p* < 0.001) but was weaker and not statistically significant in women (r = 0.240, *p* = 0.100). The post-treatment difference in correlation strength between men and women was not statistically significant (z = 1.59, *p* = 0.11). These findings suggest that LSc severity, as measured by the LSc score, is closely associated with QoL impairment, particularly among male patients.

### 3.4. LSc Score and Specific DLQI Questions

**Figure 2:**
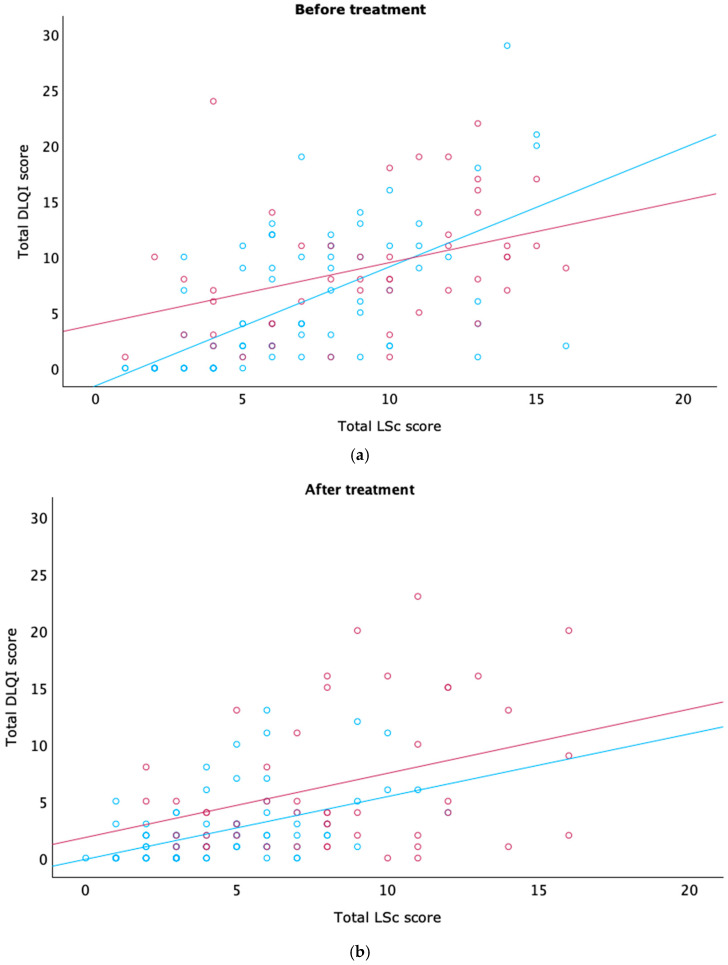
Correlation between lichen sclerosus severity and quality of life scores before and after treatment. Scatter plots showing the correlation between total Lichen Sclerosus Score (LSc score; range: 0–16) and Dermatology Life Quality Index (DLQI; range: 0–30) in male (blue) and female (red) patients (**a**) before treatment and (**b**) after 12 weeks of treatment. Stronger correlations were observed in men at both time points.

To better understand how specific aspects of quality of life are affected by LSc severity, we examined the correlation between total LSc scores and responses to DLQI questions 1, 8, and 9, both before and after treatment (Table 6 and Figure 2).

- Question 1: “Over the last week, how itchy, sore, painful or stinging has your skin been?”
- Question 8: “Over the last week, how much has your skin created problems with your partner or any of your close friends or relatives?”
- Question 9: “Over the last week, how much has your skin caused any sexual difficulties?”

In the overall cohort, significant correlations were found between LSc scores and all three questions—both before and after treatment. Among men, similar significant correlations were observed for all three items at both timepoints.

In contrast, among women, significant correlations were limited:

- Before treatment, only Question 1 (symptoms) was significantly associated with LSc severity;
- After treatment, only Question 8 (interpersonal impact) showed a significant correlation;
- No significant association was found with Question 9 (sexual difficulties) at either timepoint.

These findings suggest that clinical disease severity is more consistently aligned with QoL disruption in men than in women, particularly regarding sexual and relational domains.

### 3.5. Anatomical Site of Involvement

Anatomical sites of LSc involvement were categorized and grouped as detailed in Table 7. Patients were assigned to a site group only if disease was limited to that specific region; individuals with multi-site involvement were excluded from single-site comparisons.

Among men, the most frequently affected group was the foreskin and corona (41%). In women, the most commonly involved site was the perineum (40%). When considered individually (regardless of grouping), the most commonly affected anatomical site in men was the foreskin (85%), while in women it was the perineum (79%).

Table 7 presents the frequency and percentage of patients assigned to specific anatomical site groups for analysis. Patients were included in a group only if LSc was limited to the listed site(s); those with overlapping or multi-site involvement were excluded from these individual groupings. The most frequently affected sites were the foreskin in men and the perineum in women. n = number of patients.

**Figure 3:**
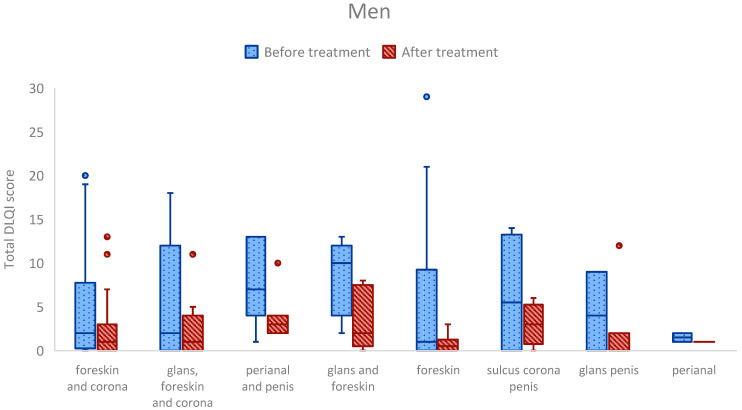

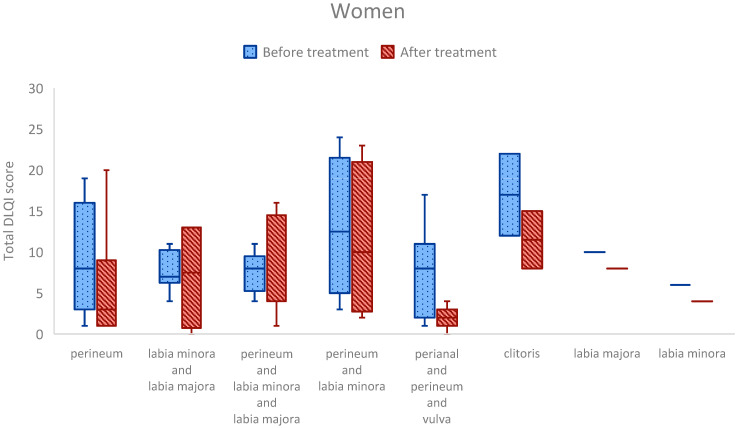
DLQI scores by anatomical site of LSc in men and women. Box plots showing total DLQI scores before and after treatment by anatomical site in men and women. Each box represents the interquartile range (25th–75th percentile), the central line indicates the median, whiskers show the non-outlier range, and dots represent outliers.

When comparing total DLQI scores across anatomical sites, no statistically significant differences were observed in either men or women after adjusting for multiple comparisons. However, some notable trends emerged. Among men, the highest pre-treatment DLQI scores (median: 10) were seen in those with LSc affecting both the glans and foreskin. In women, the highest DLQI scores (median: 17) were reported in those with isolated clitoral involvement (Figure 3).

**Figure 4:**
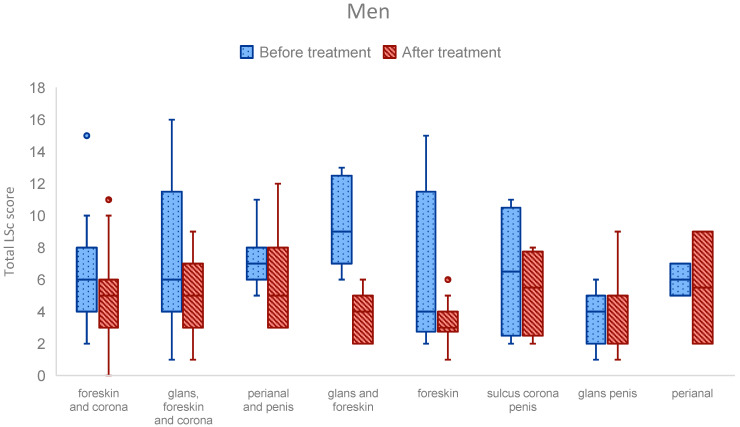

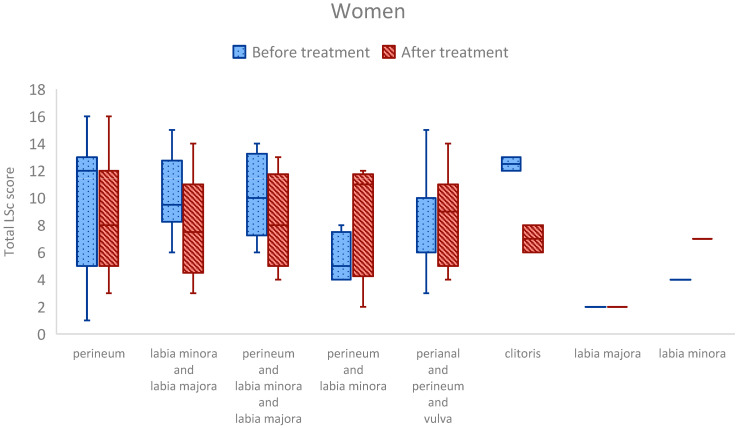
LSc scores by anatomical site of involvement in men and women. Box plots showing total LSc scores before and after the treatment by anatomical site in men and women. Central lines represent medians; boxes span the 25th to 75th percentile, with whiskers indicating the full data range excluding outliers. Though some site-specific trends were seen, none were statistically significant after correction for multiple comparisons.

Similarly, when comparing total LSc scores across anatomical sites, no significant differences remained after correction for multiple comparisons (Figure 4). Nonetheless, trends were again observed. In men, the highest median LSc score before treatment (median: 9) occurred in the group with glans and foreskin involvement. Among women, the highest median LSc score (median: 13) was noted in patients with clitoral-only disease.

### 3.6. Anatomical Site Comparisons: Clinically Relevant Trends

Although no statistically significant differences in DLQI or LSc scores between anatomical sites were observed after correction for multiple comparisons, several notable patterns emerged in unadjusted analyses.

In men, those with involvement of both the penis and perianal area reported significantly higher DLQI scores after treatment compared to those with disease confined to the foreskin (*p* = 0.002). Among women, post-treatment DLQI scores were significantly higher in patients with isolated clitoral involvement compared to those with disease affecting the perianal, perineal, and vulvar regions (*p* = 0.031).

Similarly, differences in LSc scores before treatment were observed. Men with involvement of both the glans and foreskin had higher pre-treatment LSc scores compared to those with glans-only disease (*p* = 0.003). In women, those with clitoral involvement had higher baseline LSc scores than those with disease affecting both the perineum and labia majora (*p* = 0.039).

While these findings did not retain statistical significance after multiple comparison correction, they may still be clinically meaningful. These results suggest that disease affecting highly sensitive or functional anatomical sites—such as the glans, frenulum, or perianal region in men, or the clitoris in women—may be associated with more severe symptoms and greater QoL impairment, even when lesion extent is limited.

## 4. Discussion

This prospective cohort study demonstrated a significant reduction in clinical disease severity, as measured by the LSc score, following 12 weeks of treatment in the overall cohort and in male patients. In women, although LSc scores decreased, the change did not reach statistical significance, likely due to smaller sample size and reduced statistical power. The clinical relevance of a two-point change in the LSc score remains uncertain in the absence of defined severity thresholds, highlighting the need for future score validation and stratification by severity and anatomical site.

Sex-based differences in baseline and post-treatment LSc scores were evident. Women consistently had higher scores than men, which may reflect differences in health-seeking behavior, as suggested by previous studies, or limitations in the scoring system’s ability to capture disease severity equally across sexes [22]. Notably, the LSc score does not currently adjust for anatomical differences, lesion extent, disease duration, previous treatments or impact on function, which may contribute to these discrepancies.

Our cohort included more men than women, contrasting with most LSc studies that report a female predominance. This may be due to referral patterns, with women often seen by gynecologists outside dermatology services. In a prior study from our group, both sexes showed significant improvement in DLQI after treatment [32]. In the present study, DLQI correlated strongly with clinical severity in men, both before and after treatment. Among women, this correlation was weaker and only significant at baseline. This suggests that the LSc score may better reflect disease burden in men or, alternatively, that persistent symptoms such as scarring or sexual dysfunction in women are not adequately captured by the current scoring method.

The LSc score also does not account for important contextual factors, such as sexual activity, mechanical irritation, or prior anatomical changes, which may influence symptom severity and QoL [33]. Discrepancies between clinical signs and subjective experience have previously been reported in LSc and other inflammatory skin diseases [4,21,22].

We focused on DLQI Questions 1, 8, and 9, which assess symptoms, interpersonal impact, and sexual difficulties, respectively. These domains remained strongly associated with LSc severity in men both before and after treatment, aligning with previous research showing a negative impact of genital LSc on male sexual function and relationships [24,32,34,35]. Men often underreport these issues unless asked directly, underscoring the importance of routine sexual health screening in clinical practice [36].

Among women, associations were less consistent. Only DLQI Question 1 showed a significant correlation with LSc score before treatment, and Question 8 showed significance after treatment. No correlation was found with Question 9, which may reflect the limited scope of that item relative to more comprehensive scales like the Female Sexual Function Index (FSFI). A recent 2025 study using CLISSCO and validated sexual function questionnaires found the opposite pattern: stronger correlations in women but not in men [24]. Differences in methodology, patient populations, and outcome tools may explain this contrast [24,36].

Persistent sexual dysfunction in women with LSc has been reported even after effective treatment, likely due to scarring and irreversible anatomical changes (34). This may partly explain our findings, though the absence of correlation before and after treatment suggests a more complex interaction between physical symptoms, psychological factors, and relationship dynamics.

Our study is also the first, to our knowledge, to explore the impact of anatomical site on both LSc severity and QoL. While most site-based comparisons did not reach significance after correction, several site-specific trends were clinically noteworthy. As this was an exploratory analysis, we did not apply formal corrections for multiple comparisons, and findings should be interpreted with caution. In men, involvement of the glans, frenulum, perianal area, or shaft appeared more burdensome than foreskin-only disease. In women, isolated clitoral involvement was associated with particularly high DLQI and LSc scores, supporting the idea that even small lesions in functionally and neurologically sensitive sites can have disproportionate effects on QoL.

### Strengths and Limitations

This study has several strengths. It includes both male and female participants, which is uncommon in LSc research, and spans a broad age range, improving the generalizability of findings. The use of broad inclusion criteria and the exclusion of previously circumcised men enhance external validity while minimizing anatomical confounding in the male subgroup.

The prospective design allowed for standardized baseline and follow-up assessments, with clinical evaluations and scoring performed by a single dermatologist. This minimized inter-rater variability and strengthened the internal validity of the data.

Importantly, this study is among the first to explore the influence of the anatomical site of involvement on both clinical severity and quality of life in LSc. These findings provide a foundation for future investigations into site-specific disease burden and treatment needs.

However, several limitations should be acknowledged. The LSc score used in this study, while novel and clinically practical, lacks formal validation and may not capture the full spectrum of disease severity. It assigns equal weight to reversible symptoms (e.g., pruritus) and potentially irreversible signs (e.g., sclerosis) and does not consider lesion size or distribution. This may limit its sensitivity, particularly in follow-up settings. Other research groups have similarly sought to develop sex-specific severity scales that integrate subjective and anatomical factors, reflecting a growing consensus around the need for more tailored and multidimensional approaches to LSc assessment. The LSc score, while clinically practical, is not yet validated. and this represents a key limitation of the present study. Future studies should aim to formally validate the score, including psychometric evaluation and potential sex-stratified analysis. We acknowledge that effect sizes were not computed for all group comparisons; future studies should include measures such as Cliff’s delta to enhance interpretability.

Additionally, the use of the DLQI, while widely accepted, may not fully capture the sexual or psychosocial burden of LSc, especially in women. More detailed instruments such as the FSFI (Female Sexual Function Index) and IIEF (International Index of Erectile Function) may offer better insight into specific domains of dysfunction.

This was a single-center study with a modest sample size, particularly in the female subgroup. The limited number of women reduced the statistical power and may have contributed to the lack of significant findings in this group. Larger sex-stratified cohorts are needed to validate these results. Moreover, many patients had been previously treated, and data on prior treatment duration or protocols were not collected. Due to sample size constraints and high collinearity between variables (e.g., anatomical site and sex), multivariable regression was not feasible in this cohort and should be a focus for future research. We did not explore interaction effects such as sex–anatomical site due to insufficient statistical power, but future hypothesis-driven studies should evaluate these interactions. As a result, it was not possible to adequately stratify or control for treatment response and symptom duration. Treatment regimens were individualized rather than standardized, which reflects real-world clinical practice. While individualized treatment reflects routine care, it may also introduce variability in the treatment response that could confound observed associations. This heterogeneity may influence outcome interpretation, reinforcing the need for standardized protocols in future research.

The 12-week follow-up may be insufficient to capture long-term disease trajectory, relapse rates, or persistent quality of life issues, particularly for chronic disease processes such as LSc. Longer-term studies are needed to assess sustained treatment effects and the evolution of QoL over time.

## 5. Conclusions

This study demonstrates that clinical disease severity in genital lichen sclerosus, as measured by the LSc score, significantly improves following 12 weeks of treatment in most patients, particularly men. Higher LSc scores were associated with greater impairment in quality of life, especially in domains related to symptoms, sexual difficulties, and interpersonal relationships.

To our knowledge, this is the first study to explore the association between a structured clinical severity score and DLQI in LSc. While no statistically significant differences in clinical or QoL outcomes were found based on anatomical site after adjusting for multiple comparisons, certain sites, such as the clitoris in women and the glans, frenulum, or perianal area in men, appeared to contribute disproportionately to disease burden. This highlights the nuanced and site-specific impact of LSc on patients’ lives.

Future research should focus on validating comprehensive clinical severity scores that account for lesion size, anatomical distribution, symptom reversibility, and long-term consequences such as scarring. The integration of more specific sexual function measures and longer follow-up periods will also be critical to fully capturing the lived experience of patients with LSc.

A more refined approach to disease assessment will enable earlier diagnosis, more tailored treatment, and improved quality of life outcomes, particularly important given the potential for malignant transformation in untreated cases.

## Figures and Tables

**Table 1:** Patient exclusion criteria.

Exclusion Criteria
Age < 18 years
Pregnancy
Current cancer diagnosis or treatment (except for extragenital basal cell carcinoma or squamous cell carcinoma)
Prior circumcision for LSc before inclusion
Ongoing systemic anti-inflammatory or immunomodulating treatment or its discontinuation within the past week
Ongoing systemic antibiotic use or its discontinuation within the past week
Topical treatment (antibiotics, corticosteroids, calcineurin inhibitors) on the affected area within the past week
Use of antiseptics or disinfectants on the affected area within 24 h of assessment
Inability to understand Swedish or provide informed consent

**Table 2:** Modified LSc score.

	LSc Score Visit 1	LSc Score Visit 2
Pruritus (0–2)		
Burning (0–2)		
Soreness (0–2)		
Pain during intercourse (0–2)		
Erosions (0–2)		
Sclerosis (0–2)		
Redness (0–2)		
Hypopigmentation (0–2)		
Total LSc score (0–16)		

**Table 3:** Anatomical sites assessed in the modified LSc score.

Female Patients	Male Patients
Perineum	Glans penis
Labia minora	Foreskin
Labia majora	Coronal sulcus
Clitoris	Frenulum
Perianal area	Perianal area
Multisite involvement	Multisite involvement

**Table 4:** Demographic data on the study cohort.

	Total (n = 136)	Men (n = 88)	Women (n = 48)
Median age (range)	62 (18–87)	59 (18–86)	64 (31–87)

n = number of patients.

**Table 5:** Median LSc scores before and after 12 weeks of treatment.

	Baseline LSc Score (IQR)	Follow-Up LSc Score (IQR)	Z-Score	*p*-Value
Total (n = 136)	7.0 (4.0–10.0)	5.0 (3.0–8.0)	−4.357	<0.001 *
Men (n = 88)	6.0 (4.0–9.0)	4.0 (3.0–6.0)	−4.568	<0.001 *
Women (n = 48)	10.0 (6.0–13.0)	8.0 (5.0–11.0)	−1.413	<0.158

LSc scores decreased significantly in the overall cohort and among men after treatment. No statistically significant reduction was observed in women. Scores are presented as medians with interquartile ranges. A Wilcoxon signed-rank test was used to assess changes over time. Baseline LSc score = score at baseline (range: 0–16). Follow-up LSc score = score after 12 weeks. IQR = interquartile range. n = number of patients. * Statistical significance, *p* < 0.05.

**Table 6:** Correlations between total Lichen Sclerosus Score and selected DLQI items before and after treatment, stratified by sex.

	Overall Population (n = 136)		Men (n = 88)		Women (n = 48)	
	Before R (*p*-Value)	After R (*p*-Value)	Before R (*p*-Value)	After R (*p*-Value)	Before R (*p*-Value)	After R (*p*-Value)
Q1	0.604 (<0.001)	0.512 (<0.001)	0.628 (<0.001)	0.470 (<0.001)	0.495 (<0.001)	0.194 (0.188)
Q8	0.374 (<0.001)	0.351 (<0.001)	0.487 (<0.001)	0.254 (0.017)	0.088 (0.550)	0.299 (0.039)
Q9	0.389 (<0.001)	0.241 (0.005)	0.489 (<0.001)	0.255 (0.017)	0.189 (0.199)	0.102 (0.489)

Spearman’s correlation coefficients (r) and *p*-values show associations between LSc severity and DLQI Questions 1 (symptoms), 8 (interpersonal impact), and 9 (sexual difficulties) at baseline and after 12 weeks of treatment. Stronger and more consistent correlations were observed among men. Associations in women were less robust, especially regarding sexual function. LSc score range: 0–16. Q1 = DLQI question 1: “Over the last week, how itchy, sore, painful or stinging has your skin been?” (range: 0–3). Q8 = DLQI question 8: “Over the last week, how much has your skin created problems with your partner or any of your close friends or relatives?” (range: 0–3). Q9 = DLQI question 9: “Over the last week, how much has your skin caused any sexual difficulties?” (range: 0–3). n = number of patients. R = Spearman’s rho correlation coefficient (*p*-value). *p*-values are considered significant at the 0.05 level for 2-tailed tests, and at the 0.01 level for 2-tailed tests.

**Table 7:** Anatomical sites of lichen sclerosus involvement and frequency of patients by group.

Localizations in Females	n (%)	Localizations in Males	n (%)
Perineum	19 (40)	Foreskin and corona	36 (41)
Perineum and labia minora and majora	8 (17)	Foreskin	14 (16)
Perianal and perineum and vulva	7 (15)	Glans, foreskin and corona	13 (15)
Labia minora and labia majora	6 (12)	Glans penis	7 (8)
Perineum and labia minora	4 (8)	Perianal and penis	7 (8)
Clitoris	2 (4)	Glans and foreskin	5 (6)
Labia minora	1 (2)	Sulcus corona penis	4 (4)
Labia majora	1 (2)	Perianal	2 (2)
Perineum and labia majora	0 (0)	Glans and corona	0 (0)
Perianal	0 (0)	Frenulum	0 (0)

## Data Availability

The data presented in this study are available on reasonable request from the corresponding author. The data are not publicly available due to privacy and ethical restrictions.

## Supplementary Materials

The following supporting information can be downloaded at https://www.mdpi.com/article/10.3390/medsci13030111/s1: Supplementary File S1—Dermatology Life Quality Index (DLQI).
