# Supplementary material for: Assessing Quality of Life in Genital Lichen Sclerosus: The Role of Disease Severity and Localization—A Swedish Prospective Cohort Study
Source: Med Sci (Basel). 2025 Aug 5;13(3):111. doi: 10.3390/medsci13030111 (PMC12371901; doi:10.3390/medsci13030111)
Supplement: Supplementary file 1 [file medsci-13-00111-s001.zip › medsci-3747637-supplementary.pdf]

Supplementary material

Supplementary File S1 – Dermatology Life Quality Index (DLQI)

Namn: Personnummer:

DLQI
Score: [ ]

DERMATOLOGY LIFE QUALITY INDEX (DLQI)

Syftet med detta frågeformulär är att få veta hur mycket dina hudbesvär har påverkat ditt liv DEN SENASTE VECKAN. Var snäll och kryssa i en ruta för varje fråga.

1. Hur mycket har din hud **kliat**, **svidigt**, **bränt** och **stuckit** under den senaste veckan?	Väldigt mycket<br>En hel del<br>Lite grann<br>Inte alls	❑<br>❑<br>❑<br>❑	
2. Hur **generad** eller **osäker** på Dig själv har du varit på grund av din hud under den senaste veckan?	Väldigt mycket<br>En hel del<br>Lite grann<br>Inte alls	❑<br>❑<br>❑<br>❑	
3. Hur mycket har din hud hindrat dig från att **gå och handla**, **sköta ditt hem eller trädgård** under den senaste veckan?	Väldigt mycket<br>En hel del<br>Lite grann<br>Inte alls	❑<br>❑<br>❑<br>❑	Ej tillämpligt ❑
4. Hur mycket har din hud inverkat på din **klädsel** under den senaste veckan?	Väldigt mycket<br>En hel del<br>Lite grann<br>Inte alls	❑<br>❑<br>❑<br>❑	Ej tillämpligt ❑
5. Hur mycket har din hud påverkat **sociala aktiviteter** eller fritidsaktiviteter under den senaste veckan?	Väldigt mycket<br>En hel del<br>Lite grann<br>Inte alls	❑<br>❑<br>❑<br>❑	Ej tillämpligt ❑
6. Hur mycket har din hud förhindrat dig att utöva **sport** under den senaste veckan?	Väldigt mycket<br>En hel del<br>Lite grann<br>Inte alls	❑<br>❑<br>❑<br>❑	Ej tillämpligt
7. Har din hud förhindrat dig att **arbeta** eller **studera** under den senaste veckan?	Ja<br>Nej	❑<br>❑	Ej tillämpligt ❑
Om du har svarat nej på denna fråga, hur mycket har din hud varit ett problem under **arbetet** eller **studierna**?	En hel del<br>Lite grann<br>Inte alls	❑<br>❑<br>❑	Ej tillämpligt ❑
8. Hur mycket problem har din hud förorsakat dig i relationen med din **partner** eller någon av dina **nära vänner** eller **släktingar** under den senaste veckan?	Väldigt mycket<br>En hel del<br>Lite grann<br>Inte alls	❑<br>❑<br>❑<br>❑	Ej tillämpligt ❑
9. Hur mycket **sexuella svårigheter** eller **problem** har din hud orsakat under den senaste veckan?	Väldigt mycket<br>En hel del<br>Lite grann<br>Inte alls	❑<br>❑<br>❑<br>❑	Ej tillämpligt ❑
10. Hur mycket problem har **behandlingen** av din hud givit, till exempel genom att stöka till hemma eller ta upp tid under den senaste veckan?	Väldigt mycket<br>En hel del<br>Lite grann<br>Inte alls	❑<br>❑<br>❑<br>❑	Ej tillämpligt ❑

© AY Finlay. GK Khan. April 1992. Detta får inte kopieras ulan författarens tillstånd
